# UNC45B Reduction With Aging: A Myofiber‐Intrinsic Promoting Factor for Sarcopenia

**DOI:** 10.1111/acel.70502

**Published:** 2026-04-20

**Authors:** Taiga Mishima, Taiga Nagamune, Saori Tada, Daiki Watanabe, Nao Tokuda, Takashi Yamada, Akiko Hashimoto‐Hachiya, Sayaka Higo‐Yamamoto, Kohei Kido, Noriyuki Nagaoka, Aoi Ikedo, Yuuki Imai, Ryo Fujita, Seiya Mizuno, Satoru Takahashi, Katsutaka Oishi, Satoru Ato, Riki Ogasawara

**Affiliations:** ^1^ Cellular and Molecular Biotechnology Research Institute National Institute of Advanced Industrial Science and Technology (AIST) Tsukuba Japan; ^2^ Department of Life Science and Applied Chemistry Nagoya Institute of Technology Nagoya Japan; ^3^ Department of Computational Biology and Medical Sciences, Graduate School of Frontier Sciences The University of Tokyo Kashiwa Japan; ^4^ Graduate School of Sport and Health Sciences Osaka University of Health and Sport Sciences Osaka Japan; ^5^ Graduate School of Health Sciences Sapporo Medical University Sapporo Japan; ^6^ Graduate School of Biomedical and Health Sciences Hiroshima University Hiroshima Japan; ^7^ Health and Medical Research Institute National Institute of Advanced Industrial Science and Technology (AIST) Takamatsu Japan; ^8^ Integrated Research Center for Self‐Care Technology National Institute of Advanced Industrial Science and Technology (AIST) Takamatsu Japan; ^9^ Advanced Research Center for Oral and Craniofacial Sciences, Dental School Okayama University Okayama Japan; ^10^ Division of Integrative Pathophysiology, Proteo‐Science Center, PIAS Ehime University Toon Japan; ^11^ Biochemistry, Department of oral health science, Faculty of Dental Medicine Hokkaido University Sapporo Japan; ^12^ Division of Regenerative Medicine, Transborder Medical Research Center, Institute of Medicine University of Tsukuba Tsukuba Japan; ^13^ Laboratory Animal Resource Center in Transborder Medical Research Center, Institute of Medicine University of Tsukuba Tsukuba Japan; ^14^ Department of Applied Biological Science, Graduate School of Science and Technology Tokyo University of Science Chiba Japan; ^15^ Faculty of Medical Science Nippon Sport Science University Tokyo Japan

**Keywords:** chaperone, muscle atrophy, muscle force, sarcopenia

## Abstract

Skeletal muscle mass and force decline with age, and the loss of muscle force precedes muscle atrophy. However, the underlying mechanisms remain unclear. Here, we investigated the role of the myosin co‐chaperone, uncoordinated mutant number‐45 myosin chaperone B (UNC45B), in regulating muscle mass and force. UNC45B expression decreased in mouse gastrocnemius muscle with age, particularly at 24 months old, and adeno‐associated virus vector‐mediated knockdown of *Unc45b* in 3‐month‐old mouse triceps surae muscle first reduced plantar flexor torque and then decreased gastrocnemius muscle mass. In addition, *Unc45b* knockdown in the triceps surae muscle resulted in lower bone mineral density. While maximum Ca^2+^‐activated force in mechanically skinned fibers was not affected by *Unc45b* knockdown, *Unc45b* knockdown decreased the ratio of depolarization‐induced force to the maximum Ca^2+^‐activated force. We established tamoxifen‐inducible skeletal muscle‐specific *Unc45b* knockout (*Unc45b* imKO) mice to investigate whether the muscle atrophy and weakness due to the loss of *Unc45b* impacts metabolism and behavior. We found that *Unc45b* imKO reduced muscle mass and force at a whole‐body level, but did not influence systemic glucose tolerance, insulin sensitivity, or the respiratory exchange ratio. However, *Unc45b* imKO mice reduced the amount of deeper non‐rapid eye movement sleep, locomotor activity, and body temperature during the sleep phase. We conclude that UNC45B is essential for maintaining fast‐twitch muscle mass and muscle force. In addition, *Unc45b* deficiency‐mediated muscle loss is also associated with bone fragility, decreased body temperature, and impaired sleep quality.

## Graphical abstract

Aging reduces Unc45b expression in fast‐twitch muscle, concomitant with reduction in muscle contractile capacity and muscle mass in mice. *Unc45b* deficiency initially reduces depolarization‐induced force, followed by a decline in fast‐twitch muscle mass in adult mice. Systemic muscle weakness induced by skeletal muscle specific *Unc45b* knockout causes bone fragility, decreased body temperature, and impaired sleep quality.

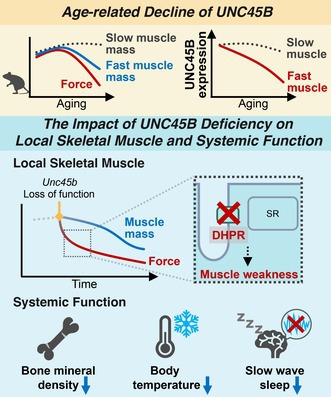

## 1 Introduction

Skeletal muscle serves many purposes in addition to body movement, including secreting regulatory factors like myokines and executing energy consumption, and skeletal muscle mass and strength are negatively associated with the risks of orthopedic disorders, cardiovascular diseases, type 2 diabetes, dementia, and mortality (Cruz‐Jentoft et al. 2019). Therefore, maintaining skeletal muscle mass and function is important to achieve healthy aging. However, even without a specific disease, skeletal muscle mass and contractile function decline with age (a.k.a. sarcopenia), and therefore understanding the mechanism is desired for the maintenance/improvement of health in old people.

Sarcopenia is characterized by a decline in muscle strength that precedes muscle atrophy, and a loss of strength that is proportionally greater than the loss of muscle mass (Ferrucci et al. 2012). These changes are observed concomitantly with the fast‐to‐slow muscle fiber type shift and preferential atrophy of fast‐muscle fibers (Larsson et al. 2019). Similarly, abnormal protein accumulation occurs preferentially in fast‐muscle fibers (Murgia et al. 2017). Although the mechanisms of those changes are not fully understood, a previous study using single muscle fiber proteomics has shown that various proteins change predominantly in human fast muscle fibers with aging (Murgia et al. 2017). These include the uncoordinated mutant number‐45 myosin chaperone B (UNC45B), and it is also observed to decrease with age in rodent muscles (Altun et al. 2010; Ato et al. 2023; Matheny et al. 2024), indicating that the decrease in UNC45B with aging is a common phenomenon in mammals.

The *unc‐45* gene was initially identified in *C. elegans* as a thick filament‐affecting gene (Brenner 1974; Epstein and Thomson 1974), and its role as a chaperone protein that maintains proper folding of the myosin motor domain was confirmed. Two paralogs of invertebrate *Unc45*, *Unc45a* and *Unc45b*, exist in vertebrates, with *Unc45a* being expressed generally in all tissues and *Unc45b* being expressed specifically in striated muscle tissue. Although the role of UNC45B in mature skeletal muscle/sarcopenia in mammals has not been investigated, it facilitates the binding of the myosin motor domain (Srikakulam et al. 2008), and the knockdown/functional loss of UNC45B results in decreases in myosin content and myofibril formation in zebrafish embryos (Bernick et al. 2010). Furthermore, a recent study reported that an exogenous *unc‐45* expression blocks age‐associated reduction in myosin and sarcomere disorganization in *C. elegans* (Matheny et al. 2024). Given the conserved role of UNC45B in myofibril assembly, a decrease in UNC45B with aging might be associated with sarcopenia in mammals. However, the role of UNC45B in the maintenance of muscle size and force in mature mammals remains unknown. Here, we aimed to investigate whether UNC45B is required in the maintenance of muscle size and force.

## 2 Materials and Methods

### 2.1 Mice

All animals were housed in an environment maintained at 22°C with a 12 h light/dark cycle (8:00 light on), with food and water *ad libitum*. All experimental procedures in this study were approved by the Ethics Committee for Animal Experiments at Nagoya Institute of Technology and AIST. Male C57BL/6J mice (Japan SLC Inc., Hamamatsu, Japan) aged 3 months old were used in *Unc45b* knockdown experiments and male C57BL/6J mice (The Jackson Laboratory Japan, Kanagawa, Japan) aged 3, 6, 9, 12, 18, and 24 months old were used in the other experiments.

### 2.2 Generation of Tamoxifen‐Inducible Skeletal Muscle‐Specific Unc45b Knockout Mice

Two mouse genomic sequences (5′‐CAG GAA GGT TCT ACC GGT AC‐3′) and (5′‐ATG CCT CAG AAC TCG GAG TG‐3′) in the intron 8 and 9 of *Unc45b* were selected as the CRISPR target. We purchased synthetic crRNA containing this target in sequence from IDT (Iowa, US). The flox donor plasmid DNA, *pflox‐Unc45b*, carried the genomic region from 1570 bp upstream to 1531 bp downstream of the exon 9 of the gene. Two loxp sequences were inserted into 628 bp upstream and 442 bp downstream of exon 9 of the gene in this donor vector. The CRISPR‐Cas9 ribonucleoprotein complex and each donor DNA were microinjected into zygotes of C57BL/6J mice (The Jackson Laboratory Japan) according to our previous report (Tanimoto et al. 2022). Subsequently, microinjected zygotes were transferred into oviducts in pseudopregnant ICR female (The Jackson Laboratory Japan) and newborns were obtained.

To confirm the designed flox allele, the genomic DNA was purified from the tail with PI‐200 (KURABO INDUSTREIS LTD, Osaka, Japan) according to the manufacturer's protocol. Genomic PCR was performed with KOD‐Fx (TOYOBO, Osaka, Japan). The primers (Unc45b flox long inLeF: 5′‐TCC TGG CAC ATA ACT CAC ACA ACT GTC G‐3′ and Unc45b flox long RiR: 5′‐GCC ATG AAG AGC TTT AAG AGC CAA AGT C‐3′) were used for detecting the flox allele. In addition, we checked for random integration of donor DNA by PCR with the primers (Donor detection‐F: 5′‐AAG GGC GAA AAA CCG TCT AT‐3′ and Donor detection‐R: 5′‐GAG ACT GGC TCA CGG AAC TC‐3′).

Inducible skeletal muscle‐specific *Unc45b* knockout (*Unc45b* imKO) mice were obtained by breeding *Unc45b‐floxed* mice with mice expressing Cre recombinase in skeletal muscle (ACTA1‐MerCreMer mice, RRID:IMSR_JAX:025750, The Jackson Laboratory, Japan). To induce recombination, 3‐ to 4‐month‐old male and female mice were administered tamoxifen (75 mg/kg body weight, S1238, Selleck Chemicals, Houston, TX, USA) dissolved in corn oil by intraperitoneal injection for five consecutive days. Tamoxifen‐treated littermate *Unc45b‐floxed* (Cre negative) mice served as control mice. Muscle mass and contractile function were assessed in both male and female mice, while all other experiments were performed exclusively in male mice.

### 2.3 Adeno‐Associated Virus Vector Injection

Three‐month‐old male C57BL/6J mice were used in UNC45B silence experiment. To silence UNC45B, adeno‐associated virus vector (AAV) serotype 6 that was expressing sh‐RNA targeting the product of the gene coding for UNC45B under cytomegalovirus promoter followed by EGFP or empty control EGFP vector was generated. The target UNC45B sequence was AGGCACTGAACCTGCTTAATA. Under isoflurane inhalation anesthesia, 5 × 10^11^ gc of AAV‐shRNA UNC45B or scramble vector in sterile saline was injected into the right and left triceps surae muscle of mice, respectively. Two, four, and 8 weeks after AAV injection, muscle force measurements were taken and triceps surae muscles were collected. All AAVs used in the present study were purchased from Vector Builder Inc. (Chicago, IL, USA).

### 2.4 Grip Test

Four limb muscle strength was measured as grip strength using a dynamometer (GPM‐101B; Melquest, Toyama, Japan). Mice were placed on a 10 × 10 cm grid with four limbs and were gently pulled backward until they released their grip. Maximum strength was calculated as the three of five consecutive measurements, excluding the maximum and minimum values.

### 2.5 In Vivo Muscle Force Measurement

Mice were immobilized on a stand under isoflurane inhalation anesthesia, and the foot was attached to a foot plate to which a force transducer was connected with the tibia‐foot angle set to 90 degrees. Surface electrodes were attached to the lower leg, and triceps surae muscle contraction was evoked via percutaneous electrical stimulation. Tetanus force (50 times of 1 ms pulse duration with 10 ms interval) was measured with optimized voltage to achieve maximal force production.

### 2.6 In Vitro Whole Muscle Force Measurement

Intact plantaris and soleus muscles were mounted with their proximal ends attached to a hook connected to a force transducer (Nihon Kohden, Tokyo, Japan) and their distal ends secured to a fixed adjustable holder. Notably, while the plantaris muscle has a well‐defined distal tendon, it has little to no proximal tendon; therefore, the proximal portion of the muscle was tied to the hook using a silk suture. Muscles were superfused with Tyrode solution bubbled with 5% CO_2_/95% O_2_ at 30°C, and were stimulated with supramaximal (50 V, 0.5 ms pulse duration) monophasic rectangular current pulses. The muscle length was adjusted to the length (*Lo*) that yielded max tetanic force. The force‐frequency relationship was determined by evoking tetani at different frequencies (1–120 Hz) at 1‐min intervals. Muscle weight and length were measured after these experiments. For the soleus, muscle length was defined as the distance between the proximal and distal myotendinous junctions. In contrast, for the plantaris, muscle length was defined as the distance from the proximal portion tied to the hook with a silk suture to the distal myotendinous junction. Force was normalized to the cross‐sectional area, calculated as the muscle weight divided by *Lo* and the density of the muscle (1056 kg/m^3^) (Yamada et al. 2009).

### 2.7 Skinned Fiber Force Measurements

#### 2.7.1 Preparation

Mechanically skinned fibers were prepared as described by Lamb and Stephenson (2018). A part of the plantaris muscle was pinned out at resting length under paraffin oil and was kept cool using an ice pack. Single muscle fibers were dissected under a stereo‐microscope and were mechanically skinned by rolling back the sarcolemma with fine forceps. Then, a segment of the skinned fiber was connected to a force transducer (Muscle tester, WPI, US), stretched to 120% of its resting length, and placed in a bath containing the standard potassium hexamethylene‐diaminetetraacetic acid (K‐HDTA) solution (see below). All skinned fiber experiments were performed at room temperature (~25°C), and Ca^2+^‐activated maximum force (Ca^2+^ force) was evaluated at the end of experiments.

#### 2.7.2 Solutions

All solutions for skinned fiber experiments were prepared as described previously (Watanabe and Wada 2025). The K‐HDTA solution was composed of 36 mM Na^+^, 126 mM K^+^, 90 mM HEPES, 8 mM ATP_total_, 1 mM free [Mg^2+^], 10 mM creatine phosphate, 0.05 mM EGTA and 50 mM HDTA, and had a pH of 7.09–7.11 at 25°C. Na‐HDTA solution was similar to the K‐HDTA solution, with all K^+^ replaced with Na^+^. The max Ca^2+^ solution was also similar to the K‐HDTA solution, but with HDTA replaced with 49.5 mM Ca‐EGTA, whereas the relaxation solution contained 50 mM free EGTA. These two solutions were mixed in an appropriate ratio with free [Ca^2+^] in the range of 10^−9^–10^−4.7^ M, and used for force–[Ca^2+^] relationship analysis (see below). Release solution was prepared by the same procedure as the K‐HDTA solution with 30 mM caffeine and 0.015 mM free [Mg^2+^] to force full opening of ryanodine receptors. Wash solutions for caffeine (WashCaf) and load‐release (WashLR) were similar to K‐HDTA except setting [Ca^2+^] at 10^−8.0^ M and 10^−7.1^ M, respectively. Load solution was similar to K‐HDTA with 10^−6.7^ M [Ca^2+^]. Caffeine solution contains 10 mM caffeine in WashCaf solution. These solutions were prepared by mixing K‐HDTA with the Relax and the max Ca^2+^ solutions at appropriate ratios (Lamb and Stephenson 2018). The 63 mM K^+^ solution is similar to K‐HDTA solution except setting [K^+^] and [Na^+^] at 63 mM and 99 mM, respectively.

#### 2.7.3 Na^+^ Depolarization and Partial Inhibition of Dihydropyridine Receptor

The skinned fiber was depolarized by replacing the K‐HDTA with the Na‐HDTA solution for ~5–6 s, which typically results in SR Ca^2+^ release and a consequent force response. This procedure was repeated until the peak force response reached a stable level. Depolarization (depol)‐induced force was evaluated relative to Ca^2+^ force (depol/Ca^2+^ force ratio). After depol‐induced force measurement, some of the fibers were exposed to 63 mM K^+^ solution for 1 min, and then Na^+^ depolarization was evoked. The depol‐induced force after the equilibration with 63 mM K^+^ solution was evaluated relative to that after the equilibration with K‐HDTA solution.

#### 2.7.4 Caffeine‐Induced Force

After all Ca^2+^ in SR was released by Release solution, the skinned fiber was exposed to WashCaf solution for 15 s. Then, Ca^2+^ was loaded to SR by exposing the fiber to Load solution for 30 s. After 15 s wash by WashCaf solution, fibers were exposed to Caffeine solution. Resultant caffeine‐induced force response was evaluated relative to Ca^2+^ force (Caffeine/Ca^2+^ force ratio).

#### 2.7.5 SR Ca^2+^ Uptake Assay

Each skinned fiber was first equilibrated for 90 s in K‐HDTA. Then, the SR was emptied of all its releasable Ca^2+^ by exposing the fibers to the Release solution. The fibers were washed in WashLR solution for 15 s and subjected to load the SR for 30 s in Load solution. After the fibers were equilibrated in WashLR solution for 15 s, all Ca^2+^ in SR was released by exposing the fiber to Release solution for 60 s. Thereafter, the fibers were washed for 15 s in WashLR solution, and the load‐release cycle was repeated with loading the SR for 3 min. The force‐time integral to the exposure to Release solution (response area) was indicative of the SR Ca^2+^ content (Lamb and Stephenson 2018). The response area after loading for 30 s was expressed as a percentage of that after loading for 3 min in the same fiber as an indicator of SR Ca^2+^ uptake ability (Watanabe and Wada 2021).

#### 2.7.6 Contractile Apparatus Analyses

Force‐Ca^2+^ concentration ([Ca^2+^]) curves were established with various [Ca^2+^] solutions (10^−6.4^, 10^−6.2^, 10^−6.0^, 10^−5.8^, 10^−5.6^, 10^−5.4^, and ~10^−4.7^ M). The force response elicited by this procedure was expressed as a percentage of the Ca^2+^ force. Ca^2+^ force was normalized to the cross‐sectional area of the fibers. The cross‐sectional area was modeled as a circular profile if the diameter was similar along the fiber segment and was calculated from an average of three width measurements.

### 2.8 Ex Vivo 2‐Deoxyglucose Uptake Measurement

Insulin stimulated 2‐deoxyglucose (2‐DG) uptake was measured in isolated extensor digitorum longus (EDL) and soleus muscles as previously described (Kjøbsted et al. 2021), with non‐labeled 2‐DG replacing the radioisotope‐labeled compound. Mice were anesthetized by intraperitoneal injection of a mixture of medetomidine, midazolam, and butorphanol (0.3, 4, and 5 mg/kg, respectively). The EDL and soleus muscles were removed and suspended in heated (30°C) and continuously gassed (95% O_2_ and 5% CO_2_) incubation chambers containing Krebs Ringer buffer supplemented with 0.1% bovine serum albumin (BSA), 38 mmol/L mannitol, and 2 mmol/L sodium pyruvate (KRB). After 10 min incubation, muscles were incubated for 30 min in KRB or KRB containing insulin (10 mU/mL). The uptake of 2‐DG was measured during the last 10 min of the 30 min incubation period by adding 1 mmol/L 2‐DG, 37 mmol/L mannitol, and 2 mmol/L sodium pyruvate. After incubation, muscles were frozen in liquid nitrogen. The accumulation of 2DG‐6‐P in muscle was assessed with several modifications to previously described procedures, integrating elements from two independent methods (Kido et al. 2025; Saito et al. 2011).

### 2.9 In Vivo 2‐Deoxyglucose Uptake Measurement

Mice fasted for 2 h were anesthetized with isoflurane and received a retro‐orbital injection of 2‐DG (170 nmol/g body weight) with or without insulin (1 mU/g body weight) dissolved in isotonic saline. Blood samples were collected from the tail immediately before and at 5 and 10 min after the injection to determine blood 2‐DG concentrations. At 10 min post‐injection, mice were euthanized, and the soleus and plantaris muscles were rapidly excised, cleaned of connective tissue, and frozen in liquid nitrogen. Blood 2‐DG and muscle 2DG‐6‐P levels were quantified using a modified protocol adapted from previously established methods (Kido et al. 2025; Saito et al. 2011).

### 2.10 Glucose Tolerance Test

After 2‐h fast (7:00–9:00), baseline blood glucose levels were measured. Glucose (2 g/kg body weight) was injected intraperitoneally into mice. Blood glucose levels were monitored until 2 h after the glucose administration.

### 2.11 Homeostatic Model Assessment for Insulin Resistance

After 12‐h fast (22:00–10:00), blood samples were collected, and baseline blood glucose levels were measured. Blood samples were centrifuged at 3000 g for 10 min at 4°C, and blood plasma samples were collected. Insulin levels in the plasma samples were measured by Mouse Insulin ELISA kit (Morinaga BioScience Inc., Kanagawa, Japan). The homeostatic model assessment for insulin resistance (HOMA‐IR) index was calculated from glucose (mmol/L) and insulin (μU/mL) levels, using the following formula: HOMA‐IR = glucose (mmol/L) × insulin (μU/mL)/22.5.

### 2.12 Respiratory Exchange Ratio Analysis

Mice were individually housed in metabolic chambers (MFD‐RQ, SHINFACTORY, Fukuoka, Japan) for 1 week to monitor fuel utilization. The first 3 days were used for acclimatization to the chamber environment, and the average of the cumulative data obtained from days 4 to 7 was analyzed. The cages were connected to a flow regulator and O_2_ and CO_2_ gas analyzers (ARCO‐2000 N, Arco Systems Inc., Chiba, Japan) which measured the volume of CO_2_ produced (VCO_2_) and the volume of O_2_ consumed (VO_2_). Respiratory exchange ratio (RER) was calculated as VCO_2_ divided by VO_2_. Carbohydrate (CHO) oxidation and fat oxidation were calculated using the following formula: CHO oxidation = 4.585 × VCO_2_ − 3.226 × VO_2_ (mg min^−1^). Fat oxidation = 1.695 × VO_2_ − 1.701 × VCO_2_ (mg min^−1^) (Péronnet and Massicotte 1991).

### 2.13 Body Composition Measurement

Whole body composition analyzer based on time domain nuclear magnetic resonance (Minispec LF50, Bruker, Billerica, MA, USA) was used to measure lean and fat mass rates until 9 weeks after tamoxifen injection. In brief, mice were placed in a plastic holder, which was then inserted into a tubular space on the side of the Minispec LF50 system. The mice were restrained in the holder throughout the scan to ensure the accuracy of measurements.

### 2.14 Core Body Temperature and Locomotor Activity

Three weeks after continuous tamoxifen injections, Nano‐tags (Kissei Comtec Co. Ltd., Nagano, Japan) were intraperitoneally implanted into mice under anesthesia with isoflurane. Locomotor activity and core body temperature were measured at five‐minute intervals and continuously recorded for 35 days. During this period, the mice were housed individually, and food intake was measured for 3 consecutive days to calculate the average daily intake. Recorder data were then analyzed using nano‐tag viewer software (Kissei Comtec Co.).

### 2.15 Sleep Recording and Analysis

The mice were anesthetized with isoflurane, then subcutaneously implanted in the back with an electroencephalogram and electromyography (EEG/EMG) transmitters (TL11M2‐F20‐EET; Data Science International, St. Paul, MN, USA) to record sleep. Two EEG electrodes were implanted above the dura (negative pole, +1.0 mm anterior, and +1.0 mm right‐lateral to the bregma; positive pole, +2.0 mm posterior, and +1.0 mm left‐lateral to the bregma), then fixed with dental cement. Two stainless steel wires were implanted into the neck muscles to collect EMG signals of muscle activity potentials. After a recovery period of at least 10 days following implantation, polygraphic EEG and EMG were continuously recorded for 72 h.

Cortical EEG and EMG signals were digitized at a sampling rate of 500 Hz and recorded using Dataquest A.R.T. (Data Sciences International Inc.). Polygraphs were automatically scored offline in 10‐s epochs divided into stages of wakefulness, REM and NREM sleep using SLEEPSIGN (Kissei Comtec Co. Ltd.) according to the standard criteria. Defined sleep–wake stages were visually examined and corrected if necessary. Power spectrum density was calculated at intervals of ~0.48 Hz, then EEG delta and theta frequency bands were set at 0.5–4.9 and 5.4–7.8 Hz, respectively.

### 2.16 Radiological Bone Mineral Density and Structure Examination

Tibial bone samples were fixed in phosphate‐buffered saline (PBS) containing 4% paraformaldehyde overnight at 4°C. The areal bone mineral density (BMD) of the isolated tibiae was measured by Dual‐energy X‐ray absorptiometry (DXA) using a bone mineral analyzer (DCS‐600EX, ALOKA, Tokyo, Japan). Micro‐computed tomography (μCT) scanning of the tibiae was performed according to the manufacturer's instructions using a Scanco Medical μCT35 System (SCANCO Medical, Brüttisellen, Switzerland) with an isotropic voxel size of 6 μm. We defined the regions of interest as 200 slices starting 300 μm distal to the proximal growth plate of the tibia. These images were used for 3D reconstruction and analysis. Structural parameters (3D) included cortical bone structure [bone area per total area (BA/TA), cortical BMD (Ct.BMD), and cortical thickness (Ct.Th)], and trabecular bone structure [bone volume per total volume (BV/TV), connective density (Conn‐Dens.), structure model index (SMI), trabecular number (Tb.N), trabecular thickness (Tb.Th), trabecular space (Tb.Sp), and trabecular BMD (Tb.BMD)] according to established guidelines from JBMR (Bouxsein et al. 2010).

### 2.17 Electron Microscope Analysis

EDL muscles were dissected and immediately fixed in PBS containing 2% paraformaldehyde for histological and ultrastructural analyses. The specimens were washed three times in PBS, then fixed in PBS containing 2% osmium tetroxide for 1 h. They were then washed three times in distilled water and dehydrated using a sequence of ethanol, acetone, and propylene oxide, and embedded in Quetol‐651 resin (Nisshin EM, Tokyo, Japan). The specimens were cross‐sectioned using an ultramicrotome (EM UC6, Leica, Vienna, Austria). The cross‐sections were coated with carbon using a carbon coater (SC‐701, Sanyu Electron, Tokyo, Japan) to prevent charging by the electron beam, and were observed using a field emission scanning electron microscope (FESEM) (JSM‐IT800 SHL, JEOL, Tokyo, Japan) at an acceleration voltage of 5 kV using an annular backscattered electron detector positioned directly below the objective lens. The images were shown in inverted contrast to facilitate observation.

### 2.18 Western Blotting

Powdered frozen skeletal muscles were homogenized in 10 volumes of homogenization buffer comprising 50 mM Tris–HCl (pH 7.5), 150 mM NaCl, 1 mM EDTA, 0.5% Triton X‐100, and protease and phosphatase inhibitor cocktail (Thermo Fisher Scientific Inc., Waltham, MA, USA). The homogenate was centrifuged at 2000 g for 3 min at 4°C; supernatants were collected, and the pellets were washed in 10 volumes of homogenization buffer and centrifuged again under the same conditions. Supernatants were removed, and pellets were homogenized in 10 volumes of radio‐immunoprecipitation assay (RIPA) buffer. Samples were diluted in sample buffer and heated at 95°C. Protein concentrations were determined using XL‐Bradford (KY‐1040, Pharma Foods Approscience Co. Ltd., Tokushima, Japan). Powdered frozen skeletal muscles obtained from *Unc45b‐floxed* and ACTA1‐MerCreMer; *Unc45b‐floxed* mice were homogenized in 10 volumes of RIPA buffer containing protease and phosphatase inhibitor. The homogenate was centrifuged at 10,000 g for 10 min at 4°C; then protein concentrations in the supernatants were determined using Protein Assay Rapid Kit Wako II (295‐78401, FUJIFILM Wako Pure Chemical, Osaka, Japan). Samples were diluted in sample buffer and heated for 5 min at 95°C. Equal amounts of protein were resolved with electrophoresis and transferred to ClearTrans SP polyvinylidene difluoride membranes (033‐22453, FUJIFILM Wako Pure Chemical). The membranes were washed in TBS containing 0.1% Tween‐20 (TBST) and blocked for 5 min at room temperature using blocking buffer (13779‐01, Nacalai Tesque, Kyoto, Japan). The membranes were washed and incubated overnight at 4°C with the following primary antibodies: UNC45B (21640‐1‐AP, Protein Tech Group Inc., Rosemont, IL, USA), UNC45A (HPA039228, Sigma‐Aldrich, MO, USA), HSP90 (ADI‐SPA‐830‐F, Enzo Life Sciences, NY, USA), Myosin (sc‐32732, Santa Cruz Biotechnology Inc., Santa Cruz, CA, USA), α‐Actin (sc‐58670, Santa Cruz Biotechnology Inc.), DHPR (MA3‐920, Thermo Fisher Scientific Inc.), STAC3 (20392‐1‐AP, Protein Tech Group Inc.), JPH1 (40‐5100, Thermo Fisher Scientific Inc.). The membranes were washed again in TBST and incubated for 90 min at room temperature with appropriate horseradish peroxidase‐conjugated secondary antibodies. Blots were visualized using chemiluminescent reagents and stained with Coomassie blue. Band intensity was quantified using Image Lab (Bio‐Rad Laboratories Inc.) and normalized by total protein band (Coomassie blue) intensity.

### 2.19 Immunohistochemistry

Frozen gastrocnemius and soleus muscles were transversely sliced into 8‐μm thick sections using cryostat. The sections were attached to MAS‐coated glass slides (Matsunami Glass, Kashiwada, Osaka, Japan) and air‐dried for 10 min. The slides were then rehydrated with PBS and blocked with 10% goat anti‐mouse IgG Fab fragment (Jackson ImmunoResearch, West Grove, PA, USA) and 5% normal goat serum in PBS‐T (0.3% Triton X‐100) for 30 min at room temperature. After sequential washing with PBS, the slides were incubated with primary antibodies diluted in 1% BSA containing PBS overnight at 4°C. The primary antibodies used in this study were as follows: mouse anti‐myosin heavy chain type I (1:50, BA‐D5, DSHB, IA, USA), anti‐myosin heavy chain type IIa (1:200, SC‐71, DSHB), anti‐myosin heavy chain type IIb (1:100, BF‐F3, DSHB), anti‐Laminin (1:200, L9393, Sigma‐Aldrich), anti‐MYH1 (1:200, M4276, Sigma‐Aldrich), and anti‐Laminin‐2 (1:400, L0663, Sigma‐Aldrich). The slides were washed three times with PBS and then incubated with the appropriate secondary antibodies diluted in 1% BSA containing PBS (1:250) for 90 min at room temperature. The secondary antibodies used in this study were as follows: Alexa Fluor 350 anti‐mouse IgG2b (A21140, Thermo Fisher Scientific Inc.), Alexa Fluor 488 anti‐mouse IgG1 (A21121, Thermo Fisher Scientific Inc.), Alexa Fluor 647 anti‐mouse IgM (A21238, Thermo Fisher Scientific Inc.), Alexa Fluor 555 anti‐rabbit IgG (#4413, CST), Alexa Fluor 488 anti‐mouse IgG (#4408, CST), and Alexa Fluor 647 anti‐rat IgG (ab150159, Abcam Limited, Cambridge, UK). The slides labeled with anti‐MYH1 and anti‐Laminin‐2 were subsequently incubated with 4′,6‐Diamidino‐2‐phenylindole dihydrochloride (DAPI, D9542, Sigma‐Aldrich) for 5 min at room temperature. The slides were washed and then mounted with VECTASHIELD Antifade Medium (H‐1900, Vector Laboratories, Burlingame, CA) and coverslips were secured with nail polish.

3–4 points in the surface and 2 points in the deep region of the gastrocnemius muscle and the whole cross‐section of soleus muscle of each section were acquired using a BioZero BZ‐X810 microscope (Keyence, Osaka, Japan) with ×10 objective lens. Regions of interest were acquired using Cellpose 2.0 (Pachitariu and Stringer 2022) (an average of 2890 and 725 fibers in the gastrocnemius and soleus muscles, respectively), and fiber type and cross‐sectional area were analyzed with Image J FIJI (ver. 2.16.0, National Institute of Health, Bethesda, MD, USA).

### 2.20 Statistical Analysis

Statistical comparison between two groups was performed by Student's *t*‐test. One‐way ANOVA and Two‐way ANOVA were used to statistically analyze the results. A Student's *t*‐test with a Benjamini–Hochberg false discovery rate was used as a post hoc comparison test, wherever applicable. Statistical analyses were performed using GraphPad Prism 10 (GraphPad Software Inc., La Jolla, CA, USA). Statistical significance was set at *p* < 0.05.

## 3 Results

### 3.1 Age‐Dependent Changes in Skeletal Muscle Size, Contractile Function, and UNC45B

**FIGURE 1:**
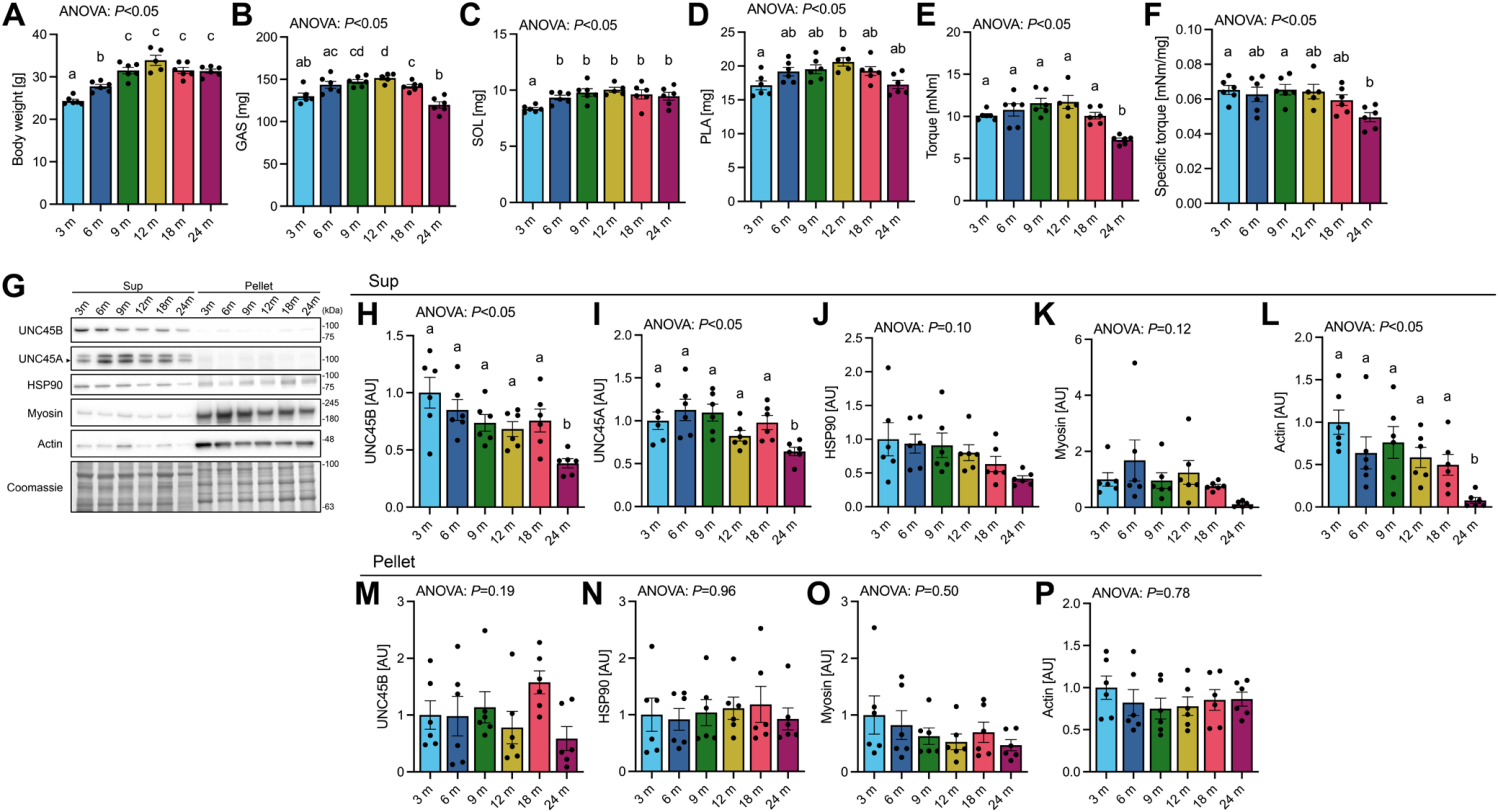
Changes in muscle mass, force, and myosin chaperone expression with age. (A) Body weight, (B) gastrocnemius, (C) soleus, and (D) plantaris muscle mass. (E) Plantar flexor torque. (F) Plantar flexor torque normalized to triceps surae muscle mass. (G) Representative western blots. Expression of (H) UNC45B, (I) UNC45A, (J) HSP90, (K) Myosin, and (L) Actin in the supernatant. Expression of (M) UNC45B, (N) HSP90, (O) Myosin, and (P) Actin in the pellet. Bar‐dot plot shows means and individual values ± standard error and individual values (3, 6, 9, 18, and 24 m, *n* = 6; 12 m, *n* = 5). Different letters indicate significant differences between groups. GAS, gastrocnemius; m, months old; PLA, plantaris; SOL, soleus; Sup, supernatant.

With the progression of aging, body weight significantly increased from 3 to 6 months and from 6 to 9 months of age but remained unchanged thereafter (Figure 1A). Gastrocnemius muscle mass was significantly higher at 9 and 12 months of age compared to 3 months (Figure 1B). Subsequently, it significantly decreased at 18 months compared to 12 months, and decreased further at 24 months (Figure 1B). Soleus muscle mass significantly increased from 3 to 6 months of age and maintained until 24 months of age (Figure 1C). Plantaris muscle mass was higher at 12 months of age compared to 3 months (Figure 1D). Electrical stimulation‐induced plantar flexor torque of the triceps surae muscle decreased at 24 months of age compared to all other age groups (Figure 1E). Specific torque also decreased at 24 months of age compared to 3 and 9 months (Figure 1F). To investigate the relationship between UNC45B expression and muscle myosin turnover, muscle samples were fractionated into supernatant (contains easily releasable myofilaments and sarcoplasmic proteins) and a pellet (contains such as myofibrillar proteins) using a low‐salt buffer (Cosper and Leinwand 2012; Neti et al. 2009; van der Westhuyzen et al. 1981). In the supernatant, expression of UNC45B in gastrocnemius and plantaris muscles but not the soleus muscle gradually declined with age, and showed a significant reduction at 24 months old compared to other age groups (Figure 1G,H, Figure S1A–C). In the supernatant, UNC45A expression levels significantly decreased at 24 months old compared to other age groups (Figure 1I). HSP90 expression levels were not altered during aging (Figure 1J). Although myosin expression appeared to be decreased at 24 months of age, this difference was not statistically significant (Figure 1K). Actin expression levels decreased at 24 months of age compared to all other age groups (Figure 1L). In the pellet, however, no age‐related differences were observed in the expression of these proteins (Figure 1M–P).

### 3.2 AAV‐Mediated Local Knockdown of Skeletal Muscle Unc45b

**FIGURE 2:**
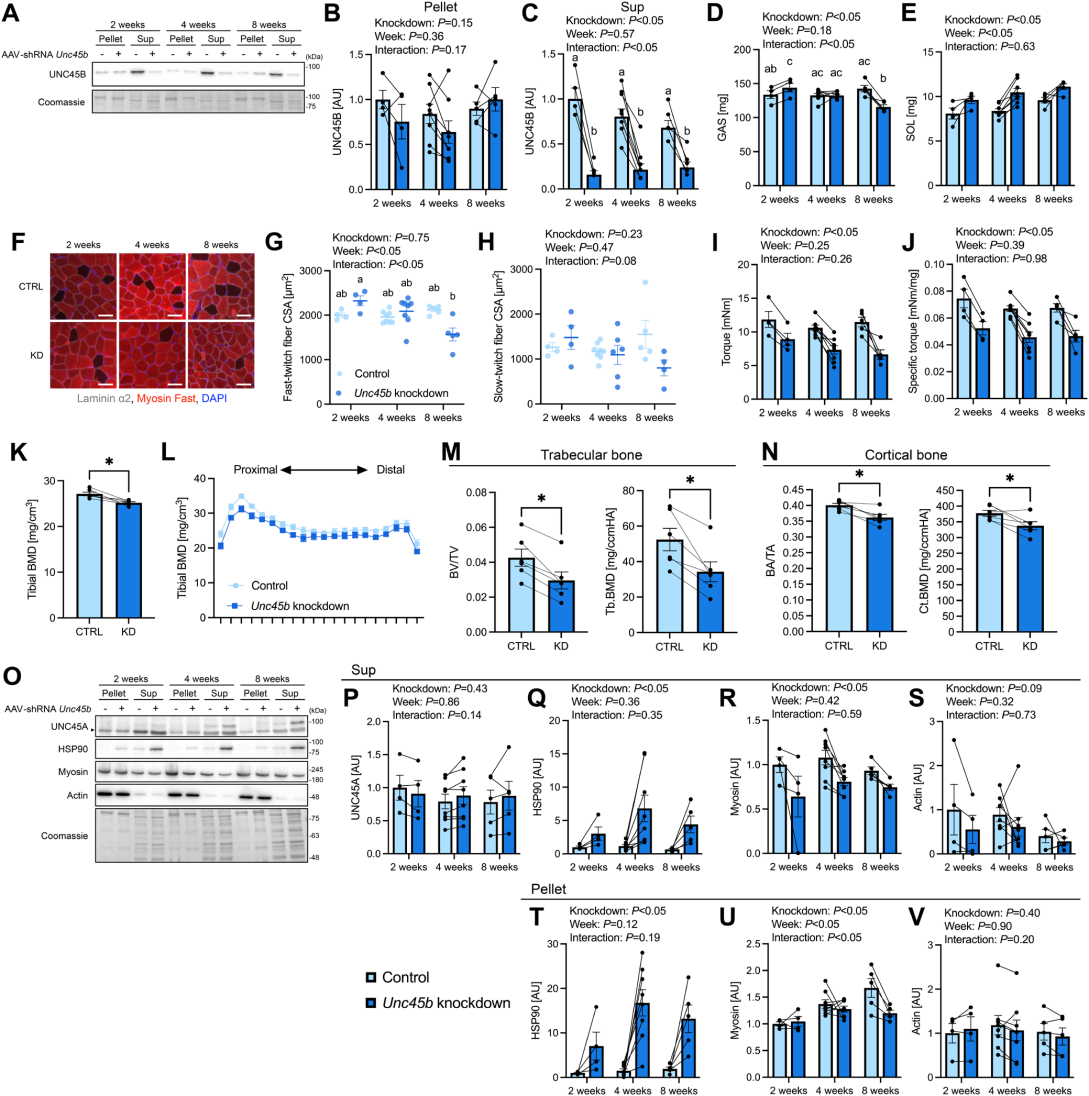
Effect of *Unc45b* knockdown in young mouse skeletal muscle on muscle mass, force, and myosin chaperone expression. (A) Representative western blots for UNC45B. Expression of UNC45B in (B) the pellet and (C) the supernatant. (D) Gastrocnemius and (E) soleus muscle mass. (F) Representative images of gastrocnemius muscle. Scale bar set to 50 μm. (G) Fast‐twitch fiber CSA. (H) Slow‐twitch fiber CSA. (I) Plantar flexor torque. (J) Plantar flexor torque normalized to triceps surae muscle mass (2 weeks, *n* = 4; 4 weeks, *n* = 7; 8 weeks, *n* = 5). (K) BMD and (L) BMD at each site when bone is divided into 20 sections. Bone area and BMD of (M) trabecular and (N) cortical bone (Control, *n* = 6; Knockdown, *n* = 6). (O) Representative western blots. Expression of (P) UNC45A, (Q) HSP90, (R) Myosin, and (S) Actin in the supernatant. Expression of (T) HSP90, (U) Myosin, and (V) Actin in the pellet (2 weeks, *n* = 4; 4 weeks, *n* = 7; 8 weeks, *n* = 5). Data are shown as means and individual values ± standard error. *p < 0.05. Different letters indicate significant differences between groups. BA, bone area; BMD, bone mineral density; BV, bone volume; CSA, cross‐sectional area; Ct, cortical; CTRL, control; GAS, gastrocnemius; KD, knockdown; SOL, soleus; Sup, supernatant; TA, total area; Tb, trabecular; TV, total volume.

To investigate the necessity of UNC45B for skeletal muscle mass and contractile function, we used an AAV‐mediated knockdown approach targeting the gene encoding UNC45B. Expression of UNC45B in the triceps surae muscle was markedly lower compared to the control leg 2 weeks after injection and this decrease persisted until 8 weeks (Figure 2A–C, Figure S2A,B). Gastrocnemius muscle mass unexpectedly increased in the knockdown leg at 2 weeks after AAV injection. This difference disappeared at 4 weeks, and at 8 weeks after AAV injection, the muscle weight was significantly lower than that of the control leg (Figure 2D). Soleus muscle mass was increased by *Unc45b* knockdown (Figure 2E). No statistically significant change in gastrocnemius muscle fiber CSA (fCSA) was observed in either fast or slow fibers (Figure 2F–H). Maximal plantar flexor torque and specific torque of the triceps surae muscle were decreased by *Unc45b* knockdown (Figure 2I,J). Isolated tibia BMD significantly decreased at 8 weeks after *Unc45b* knockdown (Figure 2K,L). Bone structure analysis by μCT revealed that *Unc45b* knockdown decreased BV/TV, Conn‐Dens, Tb.Th, Tb.BMD, BA/TA, and Ct.BMD and increased SMI, whereas Tb.N, Tb.Sp, and Ct.Th remained unchanged (Figure 2M,N, Figure S2C,D). These results suggested that *Unc45b* knockdown‐induced muscle loss caused the reduction in cortical and trabecular bone mass.

In the supernatant, the expression of UNC45A was not altered by *Unc45b* knockdown (Figure 2O,P). HSP90 expression levels were upregulated following *Unc45b* knockdown (Figure 2Q). Muscle myosin levels were decreased by *Unc45b* knockdown while Actin levels were not altered by the knockdown (Figure 2R,S). In the pellet, HSP90 expression was higher but muscle myosin expression was lower in the knockdown leg (Figure 2T,U). Expression of Actin remained unchanged (Figure 2V).

### 3.3 The Influence of Unc45b Knockdown on Contractile Function of Myofiber

**FIGURE 3:**
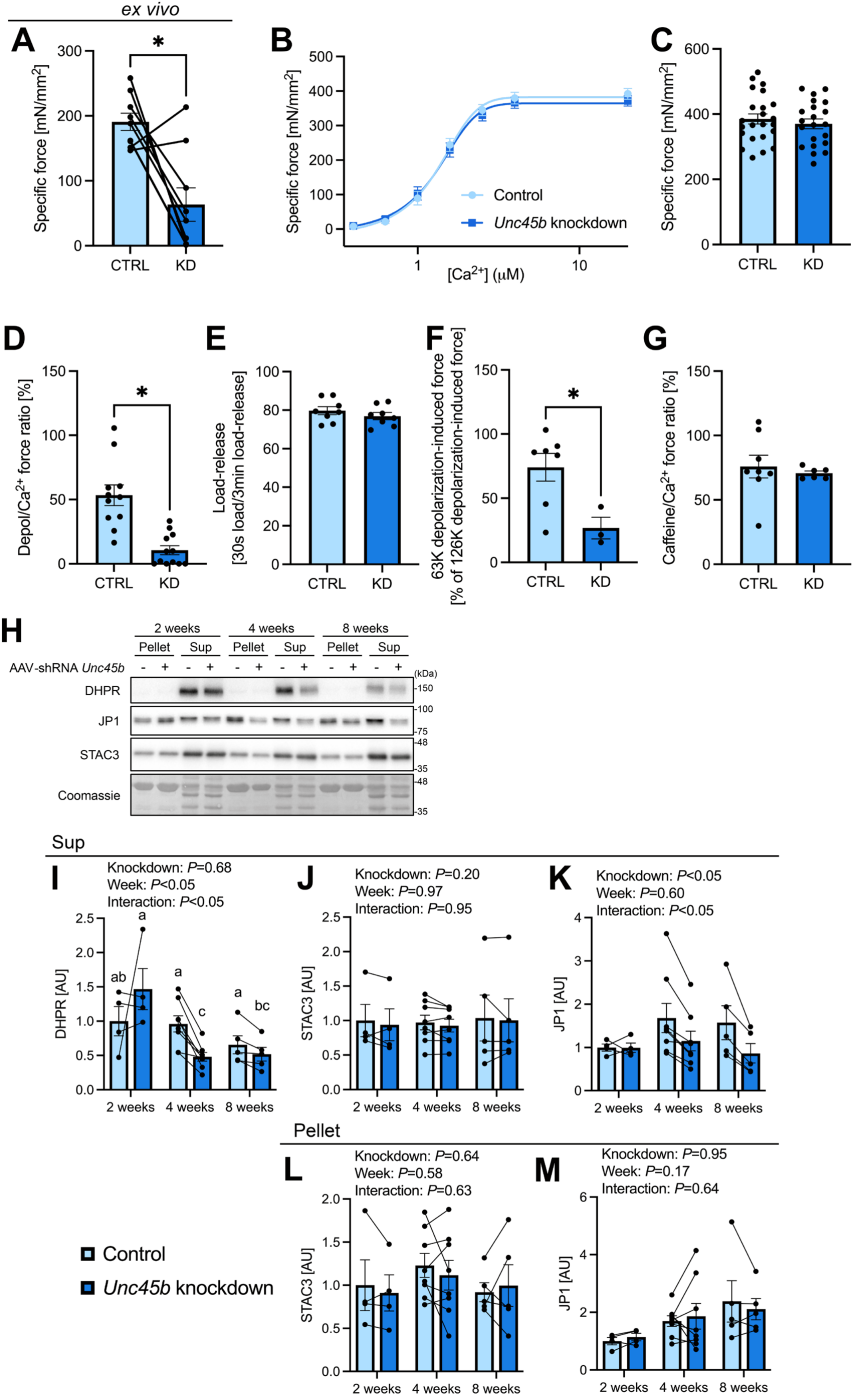
Effect of *Unc45b* knockdown in young mouse skeletal muscle on isolated plantaris muscle force and skinned fiber force. (A) Isolated plantaris muscle force normalized to plantaris muscle mass. (B) Force‐[Ca^2+^] relationship. (C) Ca^2+^ force normalized to the cross‐sectional area of the fibers (*n* = 6 muscles/group, *n* = 2–6 fibers/muscle). (D) Depolarization‐induced force (*n* = 6 muscles/group, *n* = 1–3 fibers/muscle). (E) The percentage of response area after loading for 30 s to that after loading for 3 min (*n* = 4 muscles/group, *n* = 1–3 fibers/muscle). (F) Partial depolarization‐induced force (*n* = 6 muscles/group, *n* = 1–3 fibers/muscle). (G) Caffeine‐induced force (*n* = 4 muscles/group, *n* = 1–3 fibers/muscle). (H) Representative western blots. Expression of (I) DHPR, (J) STAC3, and (K) JP1 in the supernatant. Expression of (L) STAC3, (M) JP1 in the pellet (2 weeks, *n* = 4; 4 weeks, *n* = 7; 8 weeks, *n* = 5). Data are shown as means and individual values ± standard error. *p < 0.05. Different letters indicate significant differences between groups. CTRL, control; Depol, depolarization; KD, knockdown; Sup, supernatant.

We further investigated whether the *Unc45b* knockdown‐induced reduction in muscle strength occurred within skeletal muscle. In agreement with the results of the plantar flexor torque, we found that isolated plantaris and soleus muscle force decreased 2 weeks after *Unc45b* knockdown (Figure 3A, Figure S3). Because UNC45B is known as a myosin chaperone, we hypothesized that the reduction in muscle strength caused by *Unc45b* knockdown results from impaired force production at the levels of myofibrils. We then conducted mechanically skinned fiber force measurement 2 weeks after *Unc45b* knockdown; however, the maximum Ca^2+^‐activated force was not affected by *Unc45b* knockdown (Figure 3B,C). In contrast, the Depol/Ca^2+^ force ratio was lower in the *Unc45b* knockdown group compared to the control group (Figure 3D), but no difference was found in the load‐release response, an indicator of SR Ca^2+^ uptake ability (Figure 3E). These results suggest that SR Ca^2+^ release, but not SR Ca^2+^ uptake, is impaired by *Unc45b* knockdown.

SR Ca^2+^ release function can be determined by the voltage sensor function of dihydropyridine receptor (DHPR), and the interaction between DHPR and ryanodine receptor (RyR) opening. Of these, impaired voltage sensing by DHPR likely contributes, at least in part, to the decrease in SR Ca^2+^ release, as Depol force after 63 mM K^+^ was substantially reduced (Figure 3F). The voltage sensor of DHPR can be partially inactivated in the 63 mM K^+^ solution due to membrane depolarization to ~−60 mV (Pedersen et al. 2004). In contrast, the Caffeine/Ca^2+^ force ratio was unchanged by the *Unc45b* knockdown (Figure 3G), indicating no contribution of RyR to the impaired SR Ca^2+^ release. There was no difference in DHPR level in the supernatant from the knockdown leg compared to the control leg at 2 weeks after *Unc45b* knockdown, although it decreased at 4 and 8 weeks (Figure 3H,I). This suggests that the functional impairment of DHPR observed in this study is not caused by quantitative changes in DHPR expression. Furthermore, the expression of STAC3 and JP1, which are essential for stabilizing triad junction and excitation‐contraction (EC) coupling (Golini et al. 2011; Horstick et al. 2013; Nelson et al. 2013), was unchanged at 2 weeks after *Unc45b* knockdown (Figure 3J–M), implying that the impaired SR Ca^2+^ release is not due to disrupted interaction between DHPR and RyR.

### 3.4 The Effects of Skeletal Muscle‐Specific Unc45b KO on Skeletal Muscle, Systemic Glucose Handling, and Sleep Phenotype

**FIGURE 4:**
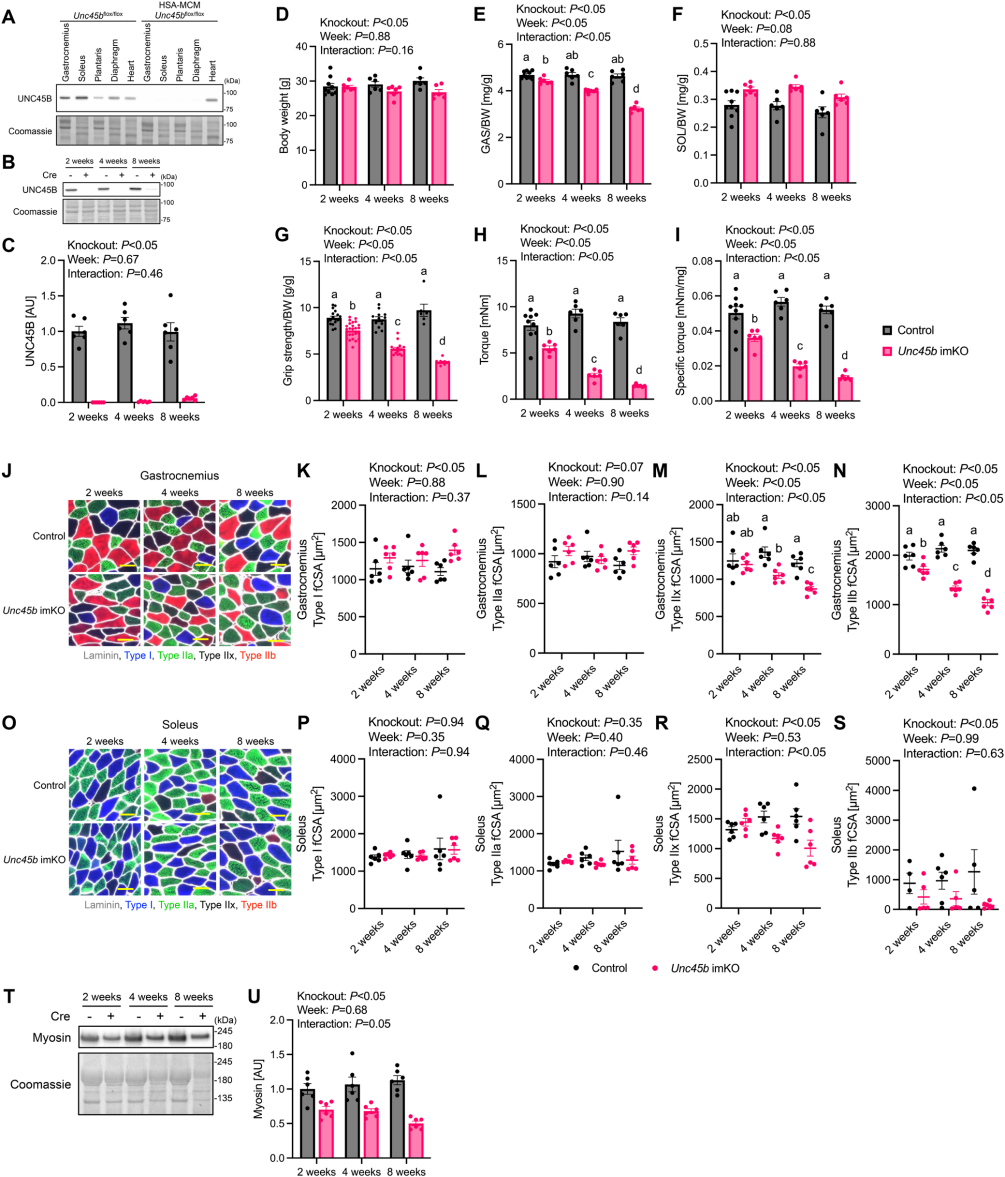
Skeletal muscle size and force characterization of tamoxifen‐inducible skeletal muscle‐specific *Unc45b* knockout mice. (A) Quantification of UNC45B expression in gastrocnemius muscle, soleus muscle, plantaris muscle, diaphragm, and heart in *Unc45b* ^*flox/flox*^ mice and ACTA1‐MerCreMer; *Unc45b* ^*flox/flox*^ mouse 8 weeks after tamoxifen injection. (B) Representative western blots for UNC45B. (C) UNC45B expression. (D) Body weight. Relative mass of the (E) gastrocnemius and (F) soleus muscles, expressed as a ratio to body weight. (G) Grip strength normalized to body weight. (H) Plantar flexor torque. (I) Plantar flexor torque normalized to triceps surae muscle mass. (J) Representative images of gastrocnemius muscle. Scale bar set to 50 μm. CSA of (K) type I, (L) type IIa, (M) type IIx, and (N) type IIb fibers in gastrocnemius muscle. (O) Representative images of soleus muscle. Scale bar set to 50 μm. CSA of (P) type I, (Q) type IIa, (R) type IIx, and (S) type IIb fibers in soleus muscle. (T) Representative western blots. (U) Myosin expression. Data are shown as means and individual values ± standard error (2 weeks, *n* = 6; 4 weeks, *n* = 6; 8 weeks, *n* = 6). Different letters indicate significant differences between groups. BW, body weight; fCSA, fiber cross‐sectional area; GAS, gastrocnemius; SOL, soleus; *Unc45b* imKO, inducible skeletal muscle‐specific *Unc45b* knockout.

We established *Unc45b* imKO mice to investigate the phenotype caused by the reduction of skeletal muscle UNC45B at the whole‐body level. Tamoxifen administration successfully decreased UNC45B expression in a skeletal muscle‐specific manner for at least 8 weeks post‐administration (Figure 4A–C). Body weight was lower in male and female *Unc45b* imKO mice (Figure 4D, Figure S4A). Gastrocnemius muscle mass normalized to body weight in male mice significantly decreased in *Unc45b* imKO mice compared to control mice at 2 weeks, and significantly declined further at 4 and 8 weeks after tamoxifen administration (Figure 4E). In female mice, gastrocnemius muscle mass normalized to body weight significantly decreased in *Unc45b* imKO mice at 4 weeks, and further reduced at 8 weeks after tamoxifen administration (Figure S4B). Soleus muscle mass normalized to body weight was increased in both males and females due to *Unc45b* imKO (Figure 4F, Figure S4C). Biceps brachii and masseter muscle mass normalized to body weight was unchanged by *Unc45b* imKO (Figure S4D,E). Maximal grip strength normalized to body weight, maximal plantar flexor torque, and specific torque of the triceps surae muscle were significantly lower in male *Unc45b* imKO mice compared to control mice at 2, 4, and 8 weeks after tamoxifen administration (Figure 4G–I). This reduction became progressively more pronounced at 4 and 8 weeks after tamoxifen administration (Figure 4G–I). In female mice, maximal grip strength normalized to body weight decreased in *Unc45b* imKO mice compared to the control mice at 2 weeks, and significantly declined further at 4 and 8 weeks after tamoxifen administration (Figure S4F). Maximal plantar flexor torque of the triceps surae muscle also decreased in female *Unc45b* imKO mice at 2 weeks and significantly worsened at 4 weeks after tamoxifen administration (Figure S4G). In the female mice, specific torque was decreased by *Unc45b* imKO (Figure S4H). In the gastrocnemius muscle, CSA of type I fibers significantly increased in *Unc45b* imKO mice (Figure 4J,K). No significant difference was observed in type IIa fibers (Figure 4L). In type IIx fibers, CSA was smaller in *Unc45b* imKO mice than in the control mice at 4 weeks, and further decreased by 8 weeks (Figure 4M). In type IIb fibers, CSA in *Unc45b* imKO mice decreased at 2 weeks compared to the control mice, and continued to decline at 4 and 8 weeks after tamoxifen administration (Figure 4N). In the soleus muscle, no significant difference was observed in CSA of type I and IIa fibers, whereas type IIx and IIb fiber CSA was lower in *Unc45b* imKO mice than in the control mice (Figure 4O–S). No significant difference was observed in fiber type composition (Figure S4I–N). UNC45A and HSP90 expression was increased by *Unc45b* imKO (Figure S4K–M). While Actin expression was unchanged, muscle myosin expression was significantly decreased by *Unc45b* imKO (Figure 4T,U, Figure S4N). Electron microscopy revealed an apparent reduction in myofibril density 9 weeks after tamoxifen administration in *Unc45b* imKO EDL muscle (Figure S4Q). These results show that *Unc45b* imKO mice have a more severe phenotype than those with AAV‐mediated *Unc45b* knockdown in triceps surae muscle.

**FIGURE 5:**
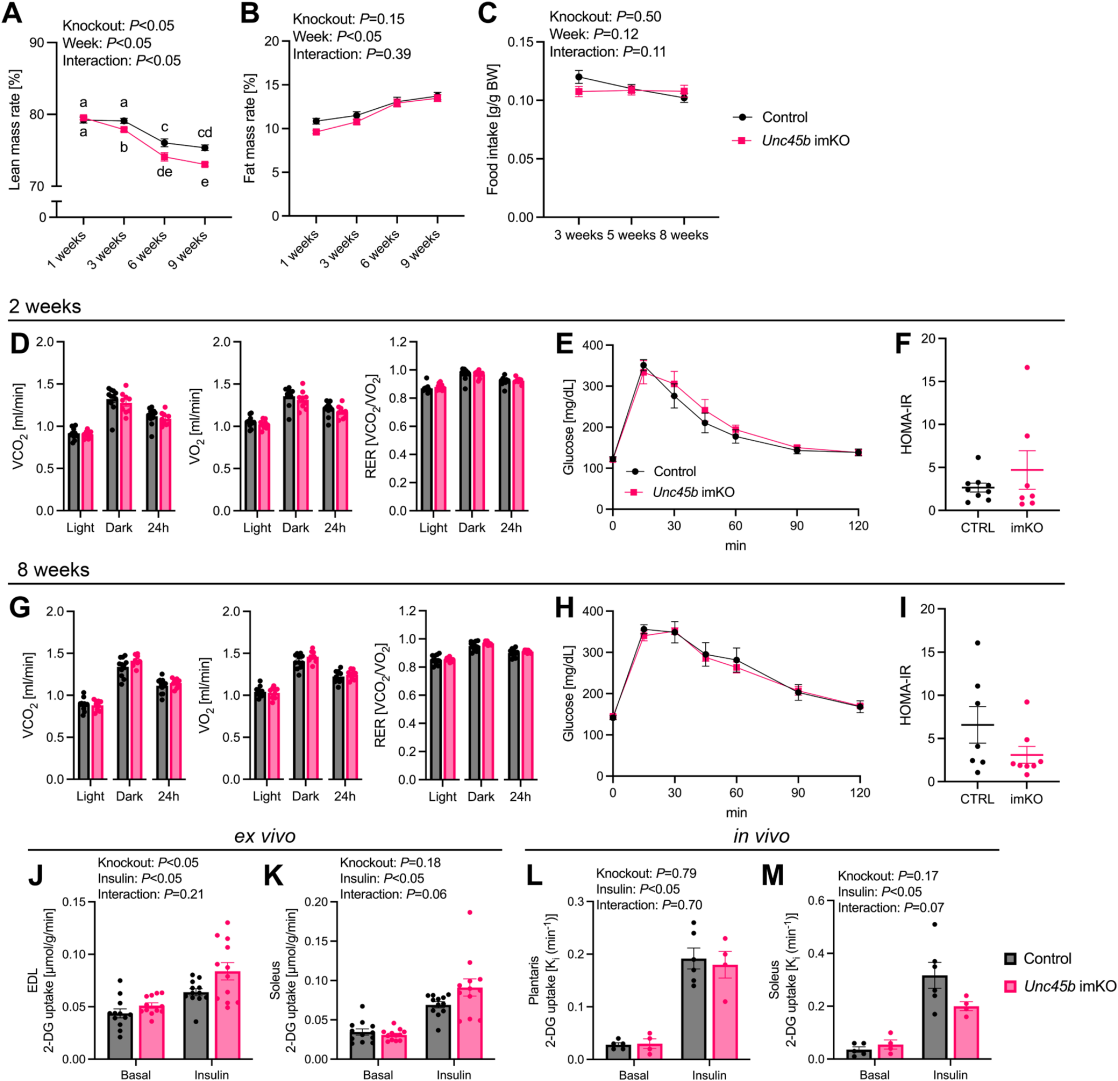
Energy and glucose metabolism of tamoxifen‐inducible skeletal muscle‐specific *Unc45b* knockout mice. (A) Lean mass rate. (B) Fat mass rate (control mice, *n* = 11; *Unc45b* imKO mice, *n* = 9). (C) Food intake in a day (control mice, *n* = 6; *Unc45b* imKO mice, *n* = 6). Average whole‐body (D) oxygen consumption, carbon dioxide production, and RER during light phase, dark phase, and all day 2 weeks after tamoxifen administration. (E) Blood glucose following an intraperitoneal glucose tolerance test 2 weeks after tamoxifen administration (control mice, *n* = 9; *Unc45b* imKO mice, *n* = 7). (F) HOMA‐IR 2 weeks after tamoxifen administration (control mice, *n* = 9; *Unc45b* imKO mice, *n* = 7). Average whole‐body (G) oxygen consumption, carbon dioxide production, and RER during light phase, dark phase, and all day 8 weeks after tamoxifen administration (control mice, *n* = 11; *Unc45b* imKO mice, *n* = 9). (H) Blood glucose following an intraperitoneal glucose tolerance test 8 weeks after tamoxifen administration (control mice, *n* = 7; *Unc45b* imKO mice, *n* = 7). (I) HOMA‐IR 8 weeks after tamoxifen administration (control mice, *n* = 7; *Unc45b* imKO mice, *n* = 8). 2‐DG uptake measured in isolated (J) EDL and (K) soleus muscle 8 weeks after tamoxifen administration (control mice, *n* = 12; *Unc45b* imKO mice, *n* = 12). In vivo 2‐DG uptake was assessed in vehicle and insulin treated mice (L) plantaris and (M) soleus muscle (control basal, *n* = 5; control insulin, *n* = 6; *Unc45b* imKO basal, *n* = 4; *Unc45b* imKO insulin, *n* = 4). Data are shown as means and individual values ± standard error. Different letters indicate significant differences between groups. EDL, extensor digitorum longus; PLA, plantaris; RER, respiratory exchange ratio; SOL, soleus; *Unc45b* imKO, inducible skeletal muscle‐specific *Unc45b* knockout.

We investigated whether the loss of muscle mass and strength caused by *Unc45b* imKO could induce metabolic alterations. Lean mass rate decreased in *Unc45b* imKO mice compared to control mice at 3, 6, and 9 weeks after tamoxifen administration, and significantly declined further at 6 and 9 weeks (Figure 5B). No significant difference in fat mass rate in *Unc45b* imKO mice was observed compared to the control mice (Figure 5B). No significant difference in food intake was observed between the control and *Unc45b* imKO mice (Figure 5C). At 2 and 8 weeks after tamoxifen administration, VO_2_, VCO_2_, RER, CHO oxidation, fat oxidation, GTT, and HOMA‐IR were comparable between the control and *Unc45b* imKO mice (Figure 5D–I, Figure S5A–D). The fasting basal blood glucose level and the plasma insulin level were also comparable between the control and *Unc45b* imKO mice (Figure S5E–H). We further examined the effect of the muscle *Unc45b* imKO on muscle glucose uptake capacity. At 8 weeks after tamoxifen administration, *Unc45b* imKO increased 2‐DG uptake in isolated EDL muscle, but the basal and insulin‐stimulated 2‐DG uptake in isolated soleus muscle were comparable between control and *Unc45b* imKO (Figure 5J,K). At 9 weeks after tamoxifen administration, there were no statistically significant differences between groups in in vivo 2‐DG uptake in plantaris and soleus muscles (Figure 5L,M).

**FIGURE 6:**
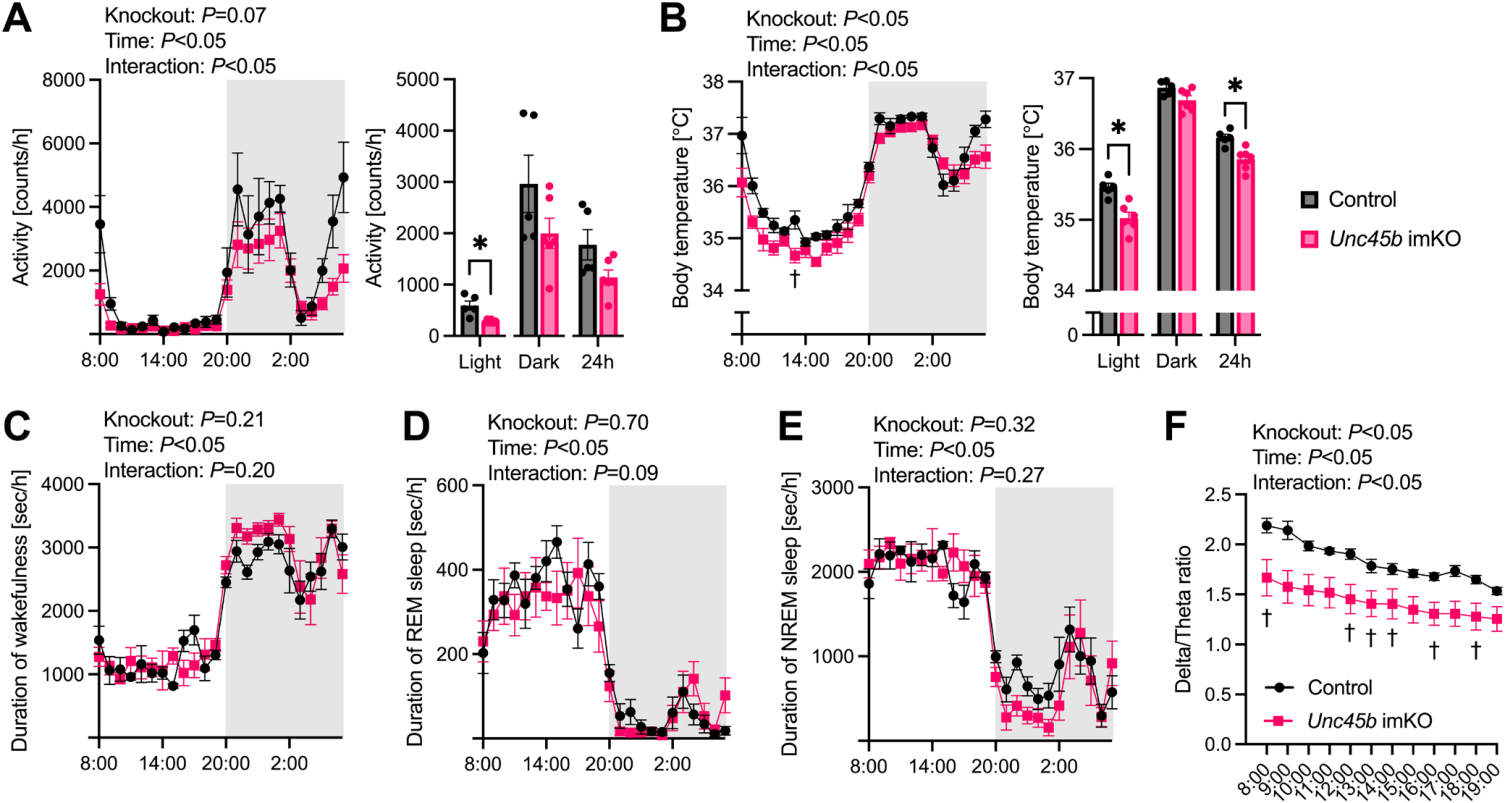
Locomotor activity, body temperature, and sleep–wake rhythms of tamoxifen‐inducible skeletal muscle‐specific *Unc45b* knockout mice. (A) Average activity during light phase, dark phase, and all day. (B) Average body temperature during light phase, dark phase, and all day (control mice, *n* = 5; *Unc45b* imKO mice, *n* = 6). Hourly time spent (C) awake, in (D) REM, and (E) NREM. (F) Hourly transition in EEG delta/theta ratio during light phase (control mice, *n* = 6; *Unc45b* imKO mice, *n* = 6). Data are shown as means and individual values ± standard error. The dark condition is highlighted in gray. *p < 0.05; ^†^ *p* < 0.05 vs. the corresponding time point in the control mice. *Unc45b* imKO, inducible skeletal muscle‐specific *Unc45b* knockout.

Finally, we investigated whether UNC45B deficiency affects mice behavior such as locomotor activity and sleep profiles. Although the total daily activity was comparable between the control and *Unc45b* imKO mice for at least 5 weeks after tamoxifen administration, the activity during the light phase decreased in *Unc45b* imKO mice (Figure 6A). The core body temperature significantly decreased throughout the day, especially during the light phase in *Unc45b* imKO mice (Figure 6B). We used EEG to assess sleep–wake rhythms in the control and *Unc45b* imKO mice 3–5 weeks after tamoxifen administration. No significant difference in the duration of wakefulness, REM sleep, and NREM sleep was observed between the control and *Unc45b* imKO mice (Figure 6C–E). On the other hand, EEG delta/theta (slow wave activity) during the light phase decreased in *Unc45b* imKO mice (Figure 6F).

## 4 Discussion

In the present study, we observed in mice that UNC45B declines with age alongside muscle contractile capacity and muscle mass. In addition, genetic inhibition of *Unc45b* in mouse skeletal muscle caused contractile dysfunction, subsequently leading to atrophy of fast‐twitch muscle fibers. Surprisingly, the decline in muscle contractile dysfunction caused by *Unc45b* knockdown was not attributable to defects at the myofibrillar level, but rather to functional impairments in EC coupling. Finally, we generated *Unc45b* imKO mice and investigated the influence of systemic reduction in muscle strength and mass on energy metabolism especially for glucose handling and sleep behaviors. We found that although *Unc45b* imKO exhibited significant muscle contractile dysfunction and type IIx and IIb fiber‐specific atrophy, systemic energy metabolism was unaffected. On the other hand, *Unc45b* imKO exhibited a decreased slow wave activity and body temperature during sleep phase.

In agreement with previous studies in human and rodent skeletal muscle (Altun et al. 2010; Ato et al. 2023; Matheny et al. 2024; Murgia et al. 2017), we found that UNC45B expression was decreased with aging in fast‐twitch fiber‐dominant muscle tissues (gastrocnemius, plantaris). This reduction in UNC45B expression corresponded with age‐related muscle loss and weakness in mice. We then performed AAV‐mediated *Unc45b* knockdown in the mouse lower leg to clarify the necessity of UNC45B for skeletal muscle mass and strength maintenance. Contrary to expectations, UNC45B deficiency reduced force production at least partially due to an impairment of voltage sensing by DHPR, while the contractile capacity of the myofibrils themselves remained intact. This impairment of DHPR may result in decreased SR Ca^2+^ release. Although the mechanisms of age‐related EC coupling dysfunction are not fully clear, DHPR dysfunction and DHPR‐RyR uncoupling have also been exhibited in aged human skeletal muscle (Boncompagni et al. 2006; Delbono et al. 1995; Jiménez‐Moreno et al. 2008; Plant and Lynch 2002; Renganathan et al. 1997). Therefore, age‐related loss of muscle force might be attributable to voltage sensing impairment mediated by the age‐related decline in UNC45B. However, since the timing of reduced contractile function does not necessarily coincide with the decrease in DHPR expression caused by *Unc45b* knockdown, it is possible that dysfunction of DHPR itself precedes the subsequent reduction in its expression levels.

Despite *Unc45b* knockdown reducing myosin levels in the soluble fraction, myofibrillar force was maintained, suggesting that the amount of myosin incorporated into myofibrils was not affected by *Unc45b* knockdown. UNC45B has classically been thought to contribute to the folding of skeletal muscle myosin during the developmental stage (Bernick et al. 2010; Etard et al. 2008; Geach and Zimmerman 2010; Lee et al. 2011; Wohlgemuth et al. 2007). However, functional analysis of UNC45B suggests that while its chaperone activity is observed in smooth muscle myosin, it may not promote the folding of skeletal muscle myosin (Liu et al. 2008). In this study, the myosin F59 antibody was used to detect myosin, and it has been reported that this antibody cross‐reacts with myosins other than skeletal muscle myosin (i.e., Myh6, Myh7) in rodents (Miller et al. 1989). Therefore, the effects caused by UNC45B deficiency may occur through the impact on myosin II proteins other than skeletal muscle myosin. Importantly, these non‐skeletal muscle myosin II proteins (cardiac and smooth muscle myosin) have been observed to be expressed at low levels within muscle fibers (Wang et al. 2015). While the specific function of these minor myosins expressed within muscle fibers remains uncertain, it is possible that UNC45B regulates muscle contractility by maintaining quality control of these minor muscle myosin II proteins.

UNC45B depletion caused myosin type IIx, IIb fiber‐specific atrophy following the decline in muscle strength. Considering that *Unc45b* knockdown decreased muscle contractile function without affecting muscle mass in the slow‐twitch fiber dominant soleus muscle, it is suggested that lowered muscle contractility does not necessarily lead to a reduction in muscle mass. Importantly, previous studies in mice have observed that DHPR knockdown induces muscle weakness and muscle fiber atrophy in fast‐twitch fiber predominant tibialis anterior muscle (Piétri‐Rouxel et al. 2009). Therefore, the functional and quantitative DHPR impairments induced by *Unc45b* knockdown may explain, at least in part, the fast‐twitch fiber atrophy. Since *Unc45b* knockdown impaired depolarization‐induced force production but not caffeine‐induced force production, it is possible that UNC45B deficient muscle impaired coupling between neural excitation and intramuscular Ca^2+^ release, which is similar to denervation. It is well known that muscle denervation is a hallmark of aging (Arnold and Clark 2023; Rowan et al. 2012), and the experimental denervation causes muscle atrophy, particularly in fast‐twitch fibers (You et al. 2021). Deterioration of the neuromuscular junction (NMJ) is thought to be one of the major causes of sarcopenia. Therefore, UNC45B deficiency could affect the post‐synaptic part of the NMJ and lead to fast‐twitch fiber‐specific muscle atrophy.

The results of local skeletal muscle knockdown of *Unc45b* suggest that the decline in skeletal muscle UNC45B leads to a sarcopenia‐like phenotype in terms of muscle mass and force. Therefore, it is considered that the whole‐body skeletal muscle *Unc45b* KO mice model could be useful to investigate the direct relationship of loss of muscle strength and mass with aging‐associated several disorders such as diabetes and/or sleep disorders. Age‐related loss of muscle mass and force has been implicated as both a cause and consequence of metabolic disorders such as type 2 diabetes mellitus (Mesinovic et al. 2019; Scott et al. 2016; Son et al. 2017; Xu et al. 2023). Thus, we examined whether the skeletal muscle‐specific *Unc45b* KO‐induced loss of muscle mass and force causes abnormal glucose homeostasis. However, the skeletal muscle‐specific *Unc45b* KO‐induced decrease in muscle force and mass did not affect glucose tolerance and insulin sensitivity at the whole‐body level. On the contrary, interestingly, glucose uptake capacity in an isolated soleus muscle remained unaltered in *Unc45b* imKO mice, but 2‐DG uptake was enhanced in the isolated EDL muscle 8 weeks after tamoxifen administration. Muscle denervation induces insulin resistance in isolated soleus muscle, particularly in the early phase; however, this insulin resistance is attenuated in the later phase (Henriksen et al. 1991; McMillin et al. 2021; Wilkes and Bonen 2000). Furthermore, long‐term denervation has been observed to enhance both basal and insulin‐stimulated glucose uptake in isolated EDL muscle (Callahan et al. 2015; McMillin et al. 2021). These previous and current observations suggest that the redundancy of glucose uptake capacity in fast‐twitch skeletal muscle under muscle atrophy with impaired neural input may serve as homeostatic regulation of systemic glucose tolerance in the *Unc45b* imKO mice. In addition, in vivo insulin‐stimulated 2‐DG uptake in the soleus muscle trended lower without reaching statistical significance at 8 weeks after tamoxifen administration. It has been reported that oxidative muscles, more so than glycolytic muscles, exhibit age‐related insulin resistance in vivo (Escriva et al. 1997; Escrivá et al. 2007; Sharma et al. 2010). Thus, it is plausible that longer‐term skeletal muscle‐specific *Unc45b* KO would lead to aging‐like insulin resistance at the skeletal muscle level, particularly in slow‐twitch muscle. However, the proportion of slow‐twitch muscle tissue in the mouse's entire body is extremely small (Schiaffino and Reggiani 2011), so its impact on systemic glucose metabolism may be negligible.

Since patients with neuromuscular disorders exhibit disorders at the level of upper and lower motor neurons, nerve roots, brachial plexus, peripheral nerves, neuromuscular junctions, or muscles, which are known to cause sleep‐disordered breathing (SDB) (Irfan et al. 2015), we measured the sleep–wake rhythm in *Unc45b* imKO mice. We found that while total sleep time and circadian rhythms of non‐REM and REM sleep were normal in UNC45B‐deficient mice, SWS during the light phase (inactivity period) was significantly reduced. Although there are different reports in mice (Panagiotou et al. 2017; Soltani et al. 2019), it is well known that SWS decreases with age in humans (Van Cauter et al. 2000), and our findings suggest that UNC45B may be involved not only in sarcopenia but also in age‐related sleep disorders.

We showed that *Unc45b* imKO did not significantly affect daily locomotor activity, at least for 5 weeks after administration, but it significantly reduced activity during the light phase, which was synchronized with a decrease in core body temperature. The decrease in activity during the light period is thought to be due to a drop in core body temperature. It has been thought that muscle tone and overt shivering in cold temperatures are different manifestations of the same phenomenon in thermoregulation. However, Lømo et al. showed that muscle tone, which occurs within the thermoneutral zone, is an independent heat‐generating system for thermoregulation that is unrelated to overt shivering and is involved in maintaining body temperature during sleep (Lømo et al. 2020). Our findings suggest that UNC45B is a molecule involved in muscle tone and may contribute to maintaining body temperature during sleep.

Osteoporosis and sarcopenia often share common underlying mechanisms and interrelated (Das et al. 2023; Gielen et al. 2023; Hirschfeld et al. 2017; Yu et al. 2022). In this study, we found that *Unc45b* knockdown caused the reduction in total BMD and cortical and trabecular bone mass. Since UNC45B is expressed specifically in striated muscle, these results suggest that *Unc45b* knockdown‐induced muscle loss and weakness caused bone loss. Age‐related systemic changes in anabolic hormones, inflammation, and inadequate nutrition are thought to be related to both osteoporosis and sarcopenia (Kirk et al. 2019). Considering that the present results were obtained by comparing the contralateral legs within the same mice, it is possible that age‐related decline in skeletal muscle UNC45B is thought to be a factor that causes the fragility of the adjacent bone due to the local loss of muscle mass and strength.

In summary, we showed that UNC45B is essential for maintaining fast‐twitch muscle mass and muscle force. Mechanistically, this reduction induces muscle weakness, which is likely attributable to an impairment in depolarization‐induced force generation, independent of changes in EC‐coupling protein expression. In addition, our observations suggest that while muscle atrophy and weakness caused by UNC45B deficiency in skeletal muscle have limited impact on systemic metabolic function, it significantly impairs bone mineral density, body temperature, and sleep quality. Our present results suggest that recovering *Unc45b* expression may represent an effective therapeutic target to counteract age‐associated loss of muscle mass and force, as has been suggested from a study in *C. elegans* (Matheny et al. 2024). Further detailed investigation is needed to determine the efficacy of UNC45B as a molecular target for sarcopenia and associated age‐related diseases.

## Author Contributions

Conceptualization: R. Ogasawara; methodology: D. Watanabe, T. Yamada, K. Kido, N. Nagaoka, A. Ikedo, Y. Imai, R. Fujita, S. Mizuno, S. Takahashi, K. Oishi, S. Ato, and R. Ogasawara; validation: T. Mishima, T. Nagamune, S. Tada, D. Watanabe, N. Tokuda, T. Yamada, A. Hachiya‐Hashimoto, S. Higo‐Yamamoto, K. Kido, N. Nagaoka, A. Ikedo, Y. Imai, R. Fujita, S. Mizuno, S. Takahashi, K. Oishi, S. Ato, and R. Ogasawara; formal analysis: T. Mishima, T. Nagamune, S. Tada, D. Watanabe, N. Tokuda, T. Yamada, A. Hachiya‐Hashimoto, S. Higo‐Yamamoto, K. Kido, N. Nagaoka, A. Ikedo, Y. Imai, R. Fujita, S. Mizuno, S. Takahashi, K. Oishi, S. Ato, and R. Ogasawara; investigation: T. Mishima, T. Nagamune, S. Tada, D. Watanabe, N. Tokuda, T. Yamada, A. Hachiya‐Hashimoto, S. Higo‐Yamamoto, K. Kido, N. Nagaoka, A. Ikedo, Y. Imai, R. Fujita, S. Mizuno, S. Takahashi, K. Oishi, S. Ato, and R. Ogasawara; writing – original draft: T. Mishima, D. Watanabe, T. Yamada, K. Kido, N. Nagaoka, A. Ikedo, S. Mizuno, S. Higo‐Yamamoto, K. Oishi, S. Ato, and R. Ogasawara; writing – review and editing: T. Mishima, T. Nagamune, S. Tada, D. Watanabe, N. Tokuda, T. Yamada, A. Hachiya‐Hashimoto, S. Higo‐Yamamoto, K. Kido, N. Nagaoka, A. Ikedo, Y. Imai, R. Fujita, S. Mizuno, S. Takahashi, K. Oishi, and S. Ato; visualization: T. Mishima, S. Ato; supervision: K. Oishi, S. Ato, and R. Ogasawara; project administration: K. Kido, S. Ato, and R. Ogasawara; funding acquisition: R. Ogasawara.

## Funding

This work was supported by JSPS KAKENHI (grant numbers 22H03465 to R. Ogasawara and 24K02820 to S. Ato).

## Conflicts of Interest

The authors declare no conflicts of interest.

## Supporting information


**Figure S1:** UNC45B expression in aged soleus and plantaris muscle. (A) Representative western blots. UNC45B expression in (B) soleus and (C) plantaris muscle (3 m, *n* = 6; 24 m, *n* = 6). Data are shown as means and individual values ± standard error. *p < 0.05. Sup, supernatant; m, months old.

**Figure S2:** Effect of *Unc45b* knockdown on young mice soleus, plantaris muscle, and tibial bone. UNC45B expression in (A) soleus and (B) plantaris muscle. Quantitative data characterizing (C) trabecular bone structure and (D) cortical bone structure. Data are shown as means and individual values ± standard error. *p < 0.05. Conn‐Dens, connective density; Tb.N, trabecular number; Tb.Th, trabecular thickness; Tb.Sp, trabecular space; SMI, structure model index; Ct.Th, cortical thickness.

**Figure S3:** Effect of *Unc45b* knockdown in isolated soleus muscle force.

**Figure S4:** Phenotypic analysis of skeletal muscle mass, force, fiber type composition, and myosin chaperone expression in tamoxifen‐inducible skeletal muscle‐specific *Unc45b* knockout mice. (A) Body weight, (B) gastrocnemius muscle mass normalized to body weight, (C) soleus muscle mass normalized to body weight, (D) grip strength normalized to body weight, (E) plantar flexor torque, and (F) plantar flexor torque normalized to triceps surae muscle mass in female control and *Unc45b* imKO mice (*n* = 4–6, respectively). (G) Biceps brachii and (H) masseter muscle mass normalized to body weight in male control and *Unc45b* imKO mice (*n* = 5–6, respectively). Fiber type composition in (I) gastrocnemius and (J) soleus muscle. (K) Representative western blots. Expression of (L) UNC45A, (M) HSP90, and (N) Actin in gastrocnemius muscle (control mice, *n* = 6; *Unc45b* imKO mice, *n* = 6). (O) Representative electron microscope image of EDL muscles. Scale bar set to 5 μm. Data are shown as means and individual values ± standard error. Different letters indicate significant differences between groups. GAS, gastrocnemius; BW, body weight; SOL, soleus; *Unc45b* imKO, inducible skeletal muscle‐specific *Unc45b* knockout.

**Figure S5:** Metabolic phenotype of tamoxifen‐inducible skeletal muscle‐specific *Unc45b* knockout mice. Average (A) carbohydrate and (B) fat oxidation during light phase, dark phase, and all day 2 weeks after tamoxifen administration. Average (C) carbohydrate and (D) fat oxidation during light phase, dark phase, and all day 8 weeks after tamoxifen administration (control mice, *n* = 11; *Unc45b* imKO mice, *n* = 9). (E) Blood glucose and (F) plasma insulin concentration 2 weeks after tamoxifen administration (control mice, *n* = 9; *Unc45b* imKO mice, *n* = 7). (G) Blood glucose and (H) plasma insulin concentration 8 weeks after tamoxifen administration (control mice, *n* = 7; *Unc45b* imKO mice, *n* = 8). Data are shown as means and individual values ± standard error. CHO, carbohydrate; *Unc45b* imKO, inducible skeletal muscle‐specific *Unc45b* knockout.

## Data Availability

All remaining data in this article are available from the corresponding author upon reasonable request.
