# Supplementary figures and images for: UNC45B Reduction With Aging: A Myofiber‐Intrinsic Promoting Factor for Sarcopenia
Source: Aging Cell. 2026 Apr 20;25(5):e70502. doi: 10.1111/acel.70502 (PMC13096694; doi:10.1111/acel.70502)

# Supplemental figure 1

**A**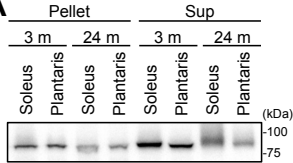**B**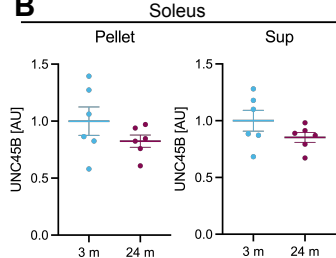**C**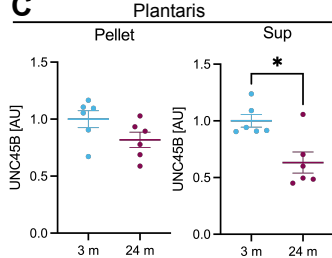

Supplement: Supplementary file 1 — Figure S1: UNC45B expression in aged soleus and plantaris muscle. (A) Representative western blots. UNC45B expression in (B) soleus and (C) plantaris muscle (3 m, n = 6; 24 m, n = 6). Data are shown as means and individual values ± standard error. *p < 0.05. Sup, supernatant; m, months old. [file ACEL-25-e70502-s002.pdf]

# Supplemental figure2

**A**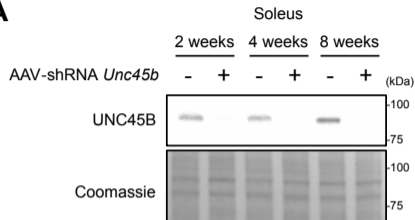**B**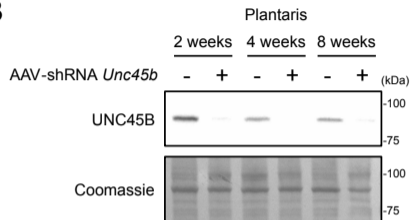**C**

Trabecular bone

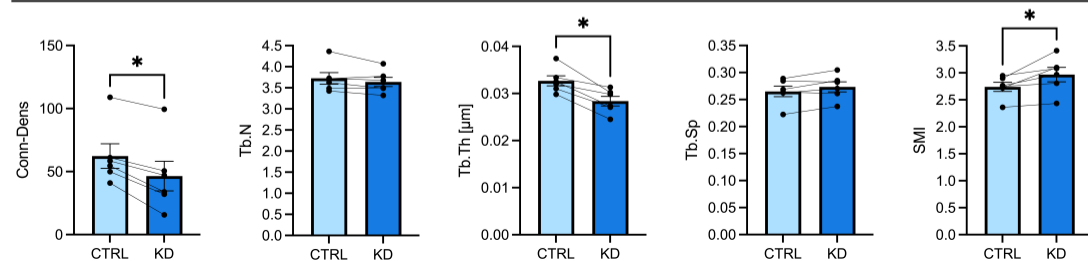**D**

Cortical bone

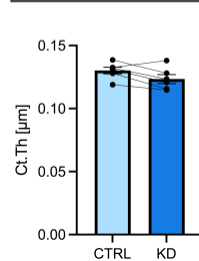

Supplement: Supplementary file 2 — Figure S2: Effect of Unc45b knockdown on young mice soleus, plantaris muscle, and tibial bone. UNC45B expression in (A) soleus and (B) plantaris muscle. Quantitative data characterizing (C) trabecular bone structure and (D) cortical bone structure. Data are shown as means and individual values ± standard error. *p < 0.05. Conn‐Dens, connective density; Tb.N, trabecular number; Tb.Th, trabecular thickness; Tb.Sp, trabecular space; SMI, structure model index; Ct.Th, cortical thickness. [file ACEL-25-e70502-s001.pdf]

# Supplemental figure3

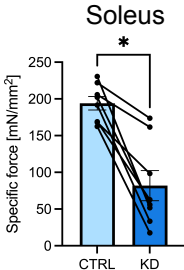

Supplement: Supplementary file 3 — Figure S3: Effect of Unc45b knockdown in isolated soleus muscle force. [file ACEL-25-e70502-s004.pdf]

# Supplemental figure4

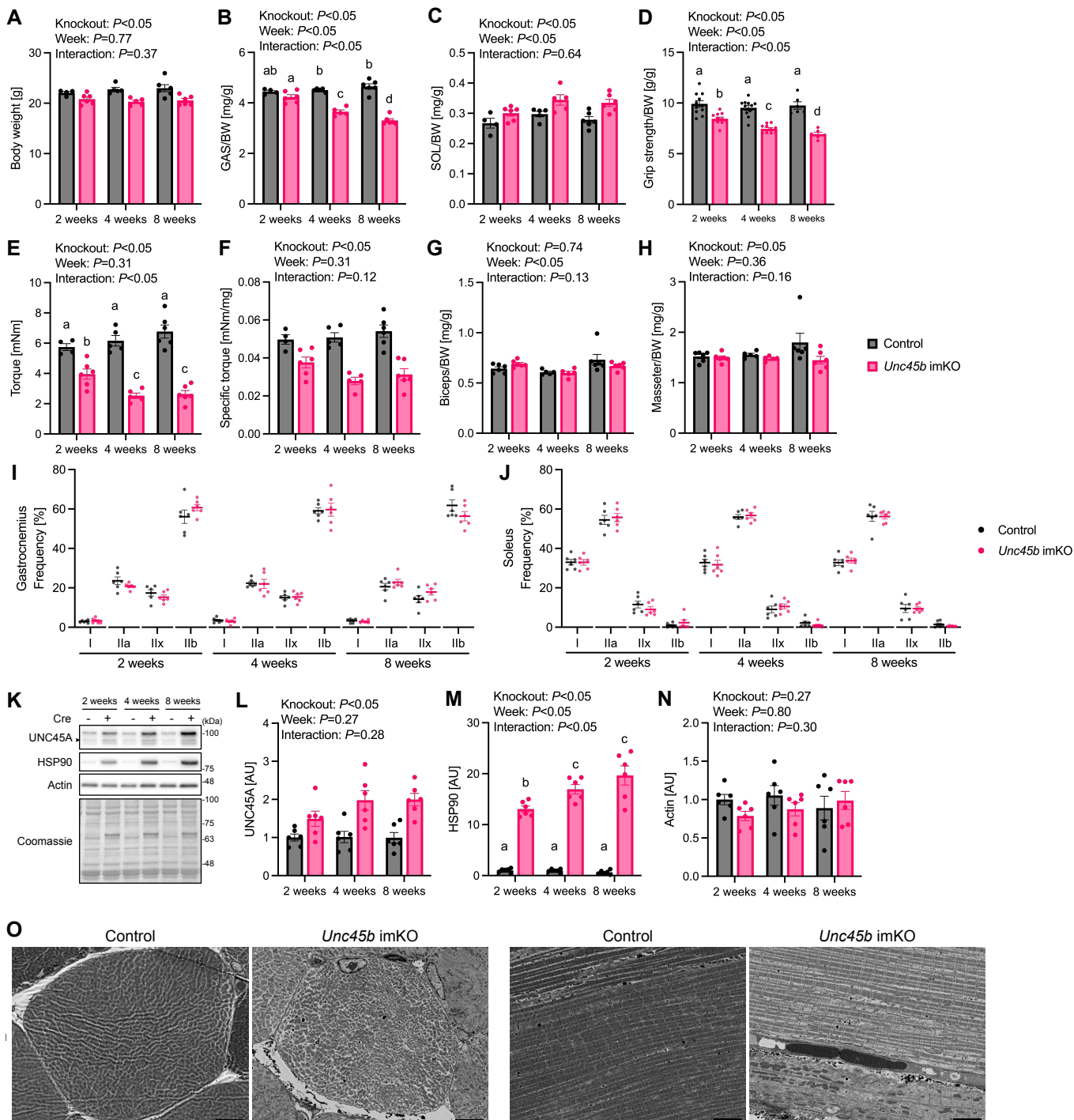

Supplement: Supplementary file 4 — Figure S4: Phenotypic analysis of skeletal muscle mass, force, fiber type composition, and myosin chaperone expression in tamoxifen‐inducible skeletal muscle‐specific Unc45b knockout mice. (A) Body weight, (B) gastrocnemius muscle mass normalized to body weight, (C) soleus muscle mass normalized to body weight, (D) grip strength normalized to body weight, (E) plantar flexor torque, and (F) plantar flexor torque normalized to triceps surae muscle mass in female control and Unc45b imKO mice (n = 4–6, respectively). (G) Biceps brachii and (H) masseter muscle mass normalized to body weight in male control and Unc45b imKO mice (n = 5–6, respectively). Fiber type composition in (I) gastrocnemius and (J) soleus muscle. (K) Representative western blots. Expression of (L) UNC45A, (M) HSP90, and (N) Actin in gastrocnemius muscle (control mice, n = 6; Unc45b imKO mice, n = 6). (O) Representative electron microscope image of EDL muscles. Scale bar set to 5 μm. Data are shown as means and individual values ± standard error. Different letters indicate significant differences between groups. GAS, gastrocnemius; BW, body weight; SOL, soleus; Unc45b imKO, inducible skeletal muscle‐specific Unc45b knockout. [file ACEL-25-e70502-s005.pdf]

# Supplemental figure5

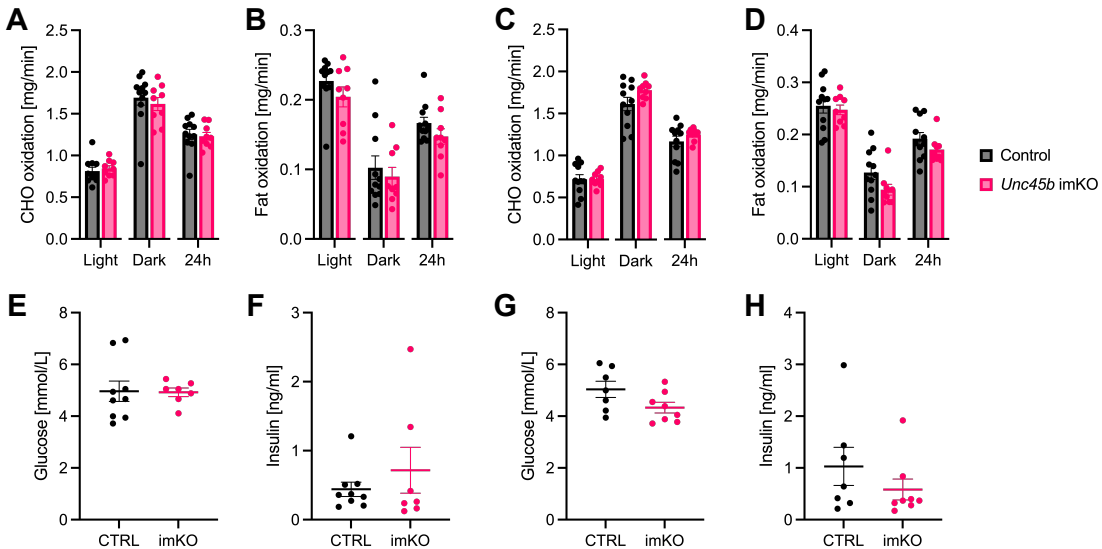

Supplement: Supplementary file 5 — Figure S5: Metabolic phenotype of tamoxifen‐inducible skeletal muscle‐specific Unc45b knockout mice. Average (A) carbohydrate and (B) fat oxidation during light phase, dark phase, and all day 2 weeks after tamoxifen administration. Average (C) carbohydrate and (D) fat oxidation during light phase, dark phase, and all day 8 weeks after tamoxifen administration (control mice, n = 11; Unc45b imKO mice, n = 9). (E) Blood glucose and (F) plasma insulin concentration 2 weeks after tamoxifen administration (control mice, n = 9; Unc45b imKO mice, n = 7). (G) Blood glucose and (H) plasma insulin concentration 8 weeks after tamoxifen administration (control mice, n = 7; Unc45b imKO mice, n = 8). Data are shown as means and individual values ± standard error. CHO, carbohydrate; Unc45b imKO, inducible skeletal muscle‐specific Unc45b knockout. [file ACEL-25-e70502-s003.pdf]
